# Role of melatonin in enhancing arbuscular mycorrhizal symbiosis and mitigating cold stress in perennial ryegrass (*Lolium perenne* L.)

**DOI:** 10.3389/fmicb.2023.1123632

**Published:** 2023-05-22

**Authors:** Hongjian Wei, Jiajin Wang, Qi Wang, Wenyuan He, Songkai Liao, Jiahao Huang, Wentao Hu, Ming Tang, Hui Chen

**Affiliations:** State Key Laboratory of Conservation and Utilization of Subtropical Agro-Bioresources, Guangdong Laboratory for Lingnan Modern Agriculture, College of Forestry and Landscape Architecture, South China Agricultural University, Guangzhou, China

**Keywords:** arbuscular mycorrhizal fungi, cold stress, melatonin, antioxidant activity, protective molecules

## Abstract

Melatonin is a biomolecule that affects plant development and is involved in protecting plants from environmental stress. However, the mechanisms of melatonin’s impact on arbuscular mycorrhizal (AM) symbiosis and cold tolerance in plants are still unclear. In this research, AM fungi inoculation and exogenous melatonin (MT) were applied to perennial ryegrass (*Lolium perenne* L.) seedlings alone or in combination to investigate their effect on cold tolerance. The study was conducted in two parts. The initial trial examined two variables, AM inoculation, and cold stress, to investigate the involvement of the AM fungus *Rhizophagus irregularis* in endogenous melatonin accumulation and the transcriptional levels of its synthesis genes in the root system of perennial ryegrass under cold stress. The subsequent trial was designed as a three-factor analysis, encompassing AM inoculation, cold stress, and melatonin application, to explore the effects of exogenous melatonin application on plant growth, AM symbiosis, antioxidant activity, and protective molecules in perennial ryegrass subjected to cold stress. The results of the study showed that compared to non-mycorrhizal (NM) plants, cold stress promoted an increase in the accumulation of melatonin in the AM-colonized counterparts. Acetylserotonin methyltransferase (ASMT) catalyzed the final enzymatic reaction in melatonin production. Melatonin accumulation was associated with the level of expression of the genes, *LpASMT1* and *LpASMT3*. Treatment with melatonin can improve the colonization of AM fungi in plants. Simultaneous utilization of AM inoculation and melatonin treatment enhanced the growth, antioxidant activity, and phenylalanine ammonia-lyase (PAL) activity, while simultaneously reducing polyphenol oxidase (PPO) activity and altering osmotic regulation in the roots. These effects are expected to aid in the mitigation of cold stress in *Lolium perenne*. Overall, melatonin treatment would help *Lolium perenne* to improve growth by promoting AM symbiosis, improving the accumulation of protective molecules, and triggering in antioxidant activity under cold stress.

## 1. Introduction

Perennial ryegrass (*Lolium perenne* L.) is extensively used as a forage or turf crop. It has a very low probability of survival during severe winters and has a semi-lethal temperature (LT_50_) of 15°C (Mugloo et al., 2023). Hence, its growth and propagation in faraway northern areas are limited. The major objective of the perennial ryegrass extension program involves formulating effective methods to improve their resistance to cold temperatures. Low temperatures greatly threaten the growth and development of tropical and temperate plants (Zhu, 2016). Cold stress disrupts the electron transport chain, leading to an increase in reactive oxygen species (ROS) production, such as hydrogen peroxide (H_2_O_2_), superoxide anion radicals (O_2_^•−^), and hydroxyl radicals (Wang et al., 2020). Excessive ROS production can cause degradation of proteins, lipids, and nucleic acids, leading to impaired cellular functions (Dreyer and Dietz, 2018; Mittler et al., 2022). Additionally, cold stress disrupts protein or protein complex stability and reduces the activity of enzymes such as ROS scavengers, resulting in photo-inhibition, impaired photosynthesis, and significant membrane damage (Ding et al., 2019). To minimize ROS-mediated oxidative stress, plants have evolved a detoxification strategy involving a network of enzymatic and non-enzymatic antioxidants. For example, enzymatic antioxidants containing catalase (CAT) and superoxide dismutase (SOD) significantly increase the rate of scavenging excess ROS under cold stress (Che et al., 2020; Pasbani et al., 2020). Certain low-molecular-weight compounds like polyamines and proline can also mitigate low-temperature-mediated oxidative damage to plants (Saadati et al., 2021).

Melatonin (N-acetyl-5-methoxytryptamine) was first isolated from the pineal gland of bovines (Lerner et al., 1958). In plants, it is a tryptophan-derived molecule that acts as an antioxidant and protects plants from abiotic stress (Liu et al., 2022). In 1995, melatonin was also found in edible crops, such as the banana (*Musa nana* Lour.) and tomato (*Solanum lycopersicum* L.), suggesting its ubiquity among plant species (Dubbels et al., 1995). More recently, melatonin receptor-1 was detected in *Arabidopsis thaliana*, which confirmed the role of melatonin as a novel growth factor responsible for plant development and stress resistance (Wei et al., 2018; Arnao and Hernandez-Ruiz, 2019). Melatonin is produced in plants through a two-step process involving four enzymes, including tryptamine 5 hydroxylase (T5H), tryptophan decarboxylase (TDC), acetylserotonin methyltransferase (ASMT), and serotonin N-acetyltransferase (SNAT; Lee et al., 2017). The conversion of tryptophan into serotonin occurs in the first step through the activity of T5H and TDC. In the second step, serotonin is converted into melatonin by ASMT and SNAT (Back et al., 2016). ASMT, functioning as the terminal enzyme, regulates the rate-limiting step in melatonin synthesis (Park et al., 2013). Plants exposed to drought and cold stress modulate melatonin production-associated gene levels, such as those of *LpASMT1* and *LpASMT3*, for enhancing stress resistance in *L. perenne* (Fu et al., 2022). Plants with a high melatonin content or those treated with melatonin exogenously are more likely to show better development and lower stress (Gao et al., 2022).

Arbuscular mycorrhizal (AM) fungi have a symbiotic relationship with the roots of several terrestrial plants (Chaudhary et al., 2023). The association with AM fungi can enhance abiotic stress tolerance in plants exposed to heavy metal stress (Kuang et al., 2023), heat stress, and cold stress (Wei et al., 2023), etc. Zhang et al. (2020) reported that the AM fungus (AMF) *Rhizophagus irregularis* increased heavy metal (Pb) tolerance in *Medicago truncatula* by enhancing melatonin synthesis. This observation indicates that symbiotic microorganisms can regulate the endogenous melatonin in host plants to enhance their resistance to abiotic stress. However, it remains unclear whether melatonin and AMF have synergistic effects on the cold resistance of plants along with the underlying mechanisms involved.

To address this, the present study hypothesized that AM inoculation could increase melatonin accumulation in host plants at low temperatures. The application of exogenous melatonin could enhance the development of mycorrhizal plants and their cold stress resistance. Consequently, the impact of melatonin treatment on AM symbiosis and cold tolerance was evaluated based on AM colonization, antioxidant activity, and osmotic compound accumulation. Additionally, the level of melatonin accumulated in cold-stressed AM-associated plants was compared to that in non-mycorrhizal (NM) plants to evaluate the effect of AM inoculation on melatonin synthesis. The present findings provided novel insights into the effect of melatonin on AMF-associated plants.

## 2. Materials and methods

### 2.1. Plant materials, AMF inoculum, and substrate

The perennial ryegrass (cv. “remier III”) was provided by the International Grass Industry Co., Ltd., Tianjin, China. The seeds were soaked in 75% ethanol for 5 min to sterilize the surface, after which they were washed four times with distilled water. The seeds were sown in plastic pots (10 × 10 cm – height × diameter) filled with a substrate of sand:peat (1:3, v/v), comprising 5.68 g kg^−1^ organic matter, 22.01 mg kg^−1^ readily available phosphorus, 34.53 mg kg^−1^ available nitrogen, and 71.23 mg kg^−1^ rapidly available potassium. The seed sowing rate was 20 g m^−2^. The substrate and the plastic pots were sterilized for 1 h at 121°C in an autoclave for three consecutive days before planting. The seedlings were raised in a growth chamber under optimal temperature conditions [25°C/20°C (day/night)], 60% relative humidity (RH), 14/10 h (light/dark) photoperiod, and photosynthetic active radiation (PAR) of 650 mmol m^−2^ s^−1^. To keep the soil moist, the pots were irrigated in the morning and the evening during the initial stage. After the seedlings emerged, the plants were watered once a day, and an acceptable soil water content (SWC) was maintained by regulating the water drainage from the pot. The crop was trimmed every 7 days to maintain a canopy height of 12 cm. It was watered and fertilized every week using half-strength Hoagland’s nutrient solution to ensure sufficient nutrient supply (Hoagland and Arnon, 1950). The use of such a solution aids in enhancing plant growth and development under abiotic stress conditions (Sardar et al., 2023).

In this study, the AMF *Rhizophagus irregularis* (*R. irregularis*) used (Bank of Glomales in China, no. BGC BJ09) was obtained from the Forestry and Landscape Architecture College of South China Agricultural University (Guangzhou, China). *Zea mays* was used as the host plant for propagation and cultured in an autoclaved substrate (1:1 v/v vermiculite/sand) for 3 months. For the propagation of *R. irregularis* in *Z. mays* roots, 100 mL of Hoagland’s solution at 1% of the standard Pi concentration (1 mM) were applied directly every 15 days into pots to satisfy the growth requirements of *Z. mays* and maintain an appropriate low phosphorus condition for *R. irregularis*. After propagation, the inoculum obtained through sucrose gradient centrifugation was used as the spore agent (Ren et al., 2021). 14-day-old seedlings of perennial ryegrass were inoculated with around 400 spores by applying 1 mL of an aqueous solution near the root system. Each non-mycorrhizal (NM) pot received the same amount of sterilized autoclaved inoculum (15 min in an autoclave at 121°C).

### 2.2. Experimental design

The pot experiments were conducted at the Forestry and Landscape Architecture College of South China Agricultural University (Guangzhou, China). Two experiments were conducted after 45 days of root colonization by AMF. The first experiment consisted of two factors, including two temperature levels [factor 1—optimal temperature (CK) and low-temperature condition (CS)] and two AM fungi treatments [factor 2—inoculated (AM)/non-inoculated (NM)]. The low-temperature treatment (LT) was administered as per the methodology outlined by Fu et al. (2022). The plants were initially acclimated to 4°C for 24 h. Subsequently, cold stress treatment was initiated, and all plants were exposed to a temperature of −8°C for 12 h. The plants were then allowed to recover from the stress by being exposed to 12 h of thawing, first at 4°C, and then under normal growth conditions. Each treatment was maintained with four sets of replications, with each set consisting of four pots of plants.

The second experiment consisted of three factors with two temperature levels [factor 1—optimal temperature (CK) and low temperature (CS)], two AM fungi treatments [factor 2—inoculated (AM)/non-inoculated (NM)], and two melatonin treatments (factor 3—with/without exogenously applied melatonin). For each treatment, four sets of replications were maintained, with each set consisting of four pots of plants. The low-temperature treatment in the experiment was administered similarly to that in the first experiment, as described by Fu et al. (2022). The seedlings were treated with a 10 μM melatonin solution for a period of 7 days, while a control group received an equivalent volume of double-distilled water.

### 2.3. Collection of plant samples and determination of biomass

Perennial ryegrass seedlings from different treatment groups (experiments 1 and 2) were collected for analysis on days 0, 3, and 6, after the initial cold stress; four replicates per treatment (four potted plants) were sampled each time. The shoots and roots of the seedlings were separated, and the biomass of dry shoots and roots was weighed. Some parts of the roots were used to assess the effect of AM colonization. The remaining root samples were ground into a fine powder using liquid nitrogen and preserved at −80°C for further analysis. On Day 6, after exposure to cold stress, the AM fungal colonization parameters were assessed, along with plant growth indicators such as shoot height, shoot and root dry weight (DW), net photosynthetic rate (Pn), and photochemical efficiency (*Fv/Fm*).

The root samples were collected at 0, 3, 6, 9, 12, and 24 h, on the first, third, and sixth days after the initial cold stress. They were marked according to the hour followed by the day in parenthesis, such as 0, 3, 6, 9, 12, 24 h (1 day), (3 days), and (6 days), respectively. In the initial experiment, four potted plants per treatment per sampling (days 3 and 6) were used to measure the expression of four melatonin synthesis genes and the endogenous melatonin content in root samples following exposure to cold stress.

### 2.4. Determination of AM colonization

First, 5% KOH was added to fix the plant roots at 80°C for 20 min, and then, 2% HCl was added to acidify for 5 min. Following acidification, the samples were treated with 0.12% trypan blue at 80°C for 20 min. The stain was subsequently removed using a solution of glycerol and lactic acid (1:1, v/v), as per the methodology outlined by Koske and Gemma (1989). The mycorrhizal colonization rate was determined using the modified magnified gridline crossing method (Hu et al., 2020) under a light microscope (Leica Microsystems, Wetzlar, Germany) at 200 × magnification. In total, 250–300 intersections were counted for each sample.

### 2.5. Measurement of the melatonin content

Melatonin was extracted using the acetone–methanol approach (Pape and Lüning, 2006). First, 0.1 g of the root sample was extracted in the dark using the extraction mixture (5 mL; acetone: methanol: water = 89:10:1). Then, the proteins were precipitated with trichloroacetic acid (TCA). After 15 min of centrifugation of the extract (12,000 × *g*, 4°C), the supernatant was collected, and the related indices were measured. The melatonin level was determined using the plant melatonin ELISA kit following the specified protocols (ZK-P7490, Ziker Biological Technology Co., Ltd., Shenzhen, China).

### 2.6. Measurement of the net photosynthetic rate and photochemical efficiency

The photosynthetic (Pn) level was measured using a portable infrared gas analyzer Li-COR6400 (LI-COR Inc., Lincoln, NE, United States), following the methodology described in another study (Wang et al., 2022). Using a fluorescence meter (Dynamax, Houston, TX, United States) and following the method described by Oxborough and Baker (1997), the photochemical efficiency (*Fv/Fm*) was measured.

### 2.7. Determination of malonaldehyde content and electrolyte leakage

The powdered root samples were first homogenized in 1.5 mL of 5% trichloroacetic acid (TCA) and then centrifuged for 25 min at 14,000 *g*. Equal volumes of supernatants (0.5 mL) were mixed with 1 mL of 20% TCA and 0.5% thiobarbituric acid (TBA), and the resulting mixture was heated at 100°C for 30 min. The heated solution was rapidly cooled and centrifuged at 10,000 *g* for 10 min. Additionally, the absorbance (OD) of the obtained supernatants was read at 450, 532, and 600 nm. The malonaldehyde (MDA) levels were detected using the extinction coefficient of 155 mM^−1^ cm^−1^ following non-specific OD deduction at 450 nm and 600 nm (Heath and Packer, 1968). The method described by Gao (2006) was used to assess electrolyte leakage (EL) in the roots.

### 2.8. Determination of the activities of antioxidant enzymes and P5C reductase

The root samples were ground into a fine powder, followed by incubation with the enzyme extraction solution consisting of 1% polyvinylpyrrolidone, 1 mM EDTA, and 50 mM potassium phosphate buffer (4°C). Then, the samples were centrifuged for 30 min (14,000 × *g*, 4°C). The activity of CAT and SOD was measured by collecting the resulting supernatant based on the methodology outlined by Beyer and Fridovich (1987). The POD activity was measured using the method described by Amako et al. (1994). The activity of ascorbate peroxidase (APX) was determined following the procedure described by Nakano and Asada (1981). Lastly, the activity of P5C reductase (P5CR) was assessed using the methodology outlined by Madan et al. (1995).

### 2.9. Determination of the content of protective molecules

To analyze the carbohydrate content, the roots were homogenized in 100 mM phosphate buffer (pH 7.5) at 4°C, followed by centrifugation at 12,000 × g for 15 min. Additionally, the supernatant was used to determine the total soluble sugar content. In contrast, the starch content was analyzed from the pellets (residual precipitate after extraction of total soluble sugar; Magne et al., 2006). The proline level was determined following the method described by Bates et al. (1973). The content of soluble phenolics was assessed based on the methodology outlined by Swain and Hillis (1959), while the content of total flavone was determined using the method described by Jia et al. (1999).

### 2.10. Measurement of phenylalanine ammonia-lyase and polyphenol oxidase activity

The root samples were ground into a fine powder and incubated with an enzyme extraction solution consisting of 1% polyvinylpyrrolidone, 1 mM EDTA, and 50 mM potassium phosphate buffer (4°C), followed by centrifugation at 14,000 × g for 30 min at 4°C.

phenylalanine ammonia-lyase (PAL) activity was measured by extracting it in a buffer solution containing 50 mM sodium borate (pH 7.0), 2 mM EDTA, 18 mM 2-mercaptoethanol, and 2% (w/v) insoluble polyvinylpyrrolidone. The resulting enzyme extract was mixed with 100 mM borate buffer (pH 8.8) and 12 mM L-phenylalanine, followed by incubation for 30 min at 30°C. The absorbance was recorded at 290 nm and the amount of trans-cinnamic acid was calculated using its extinction coefficient of 9,630 M^−1^. The enzyme activity was expressed as the rate of conversion of L-phenylalanine to trans-cinnamic acid mg^−1^ protein min^−1^ (Hajiboland et al., 2013).

Polyphenol oxidase (PPO) was extracted in 200 mM sodium phosphate buffer (pH 6.5). The assay solution consisted of 10 mM pyrogallol and 200 mM sodium phosphate buffer (pH 6.5). To initiate the reaction, the enzyme extract was added at 30°C, and absorbance changes at 334 nm were measured for 10 min to monitor pyrogallol oxidation. PAL activity was then calculated as U mg^−1^ protein min^−1^, based on the methodology described by Hajiboland et al. (2013).

### 2.11. RNA extraction and quantitative real-time PCR assay

TRIzol reagent (Life Technologies, Grand Island, NY, United States) was used for extracting the total RNA from the leaf. Next, TURB DNA-free™ reagent (Life Technologies) was used for removing the contaminated genomic DNA (gDNA) from the RNA extraction solution. The cDNA was prepared from 2 μg of total RNA by reverse transcription using a High-Capacity cDNA Reverse Transcription Kit (Life Technologies, Grand Island, NY, USA). PCR was conducted using the StepOnePlus Real-Time PCR System (Life Technologies, Grand Island, NY, United States), and gene expression levels were measured with the Power SYBR® Green PCR Master Mix (Applied Biosystems, Foster City, CA, United States). The gene-specific primers of the four melatonin-production-associated genes, *LpP5CS*, *LpPAL*, and *LpPPO*, are shown in Supplementary Table S1. The expression of the target genes was normalized against the reference gene, *LpeIHF4A* (Huang et al., 2014). Each PCR protocol was conducted with two technical and four biological replicates.

### 2.12. Statistical analysis

The statistical analysis package SPSS 22.0 (SPSS Inc., Chicago, IL, United States) was used to perform statistical analyses. The homogeneity and normality of the data were verified. The data used in the statistical analyses were found to be normally distributed. For the data obtained from experiment 1, which had two factors (i.e., inoculated AM fungi treatment and temperature conditions), multifactor ANOVA was performed to analyze the results. Fisher’s LSD test was conducted to measure the significance of the difference obtained through ANOVA. Multifactor ANOVA was also performed to analyze the data from experiment 2, which had three factors (i.e., inoculated AM fungi treatment, temperature conditions, and melatonin application). Fisher’s LSD test was conducted to determine significant differences in the ANOVA. Pearson’s test was conducted for correlation analysis (*p* < 0.05). Based on the factor analysis, which was conducted after the sphericity test of KMO and Bartlett, the principal component analysis (PCA) of all tested parameters related to antioxidants and protecting molecules was performed.

## 3. Results

### 3.1. Melatonin levels

**Figure 1:**
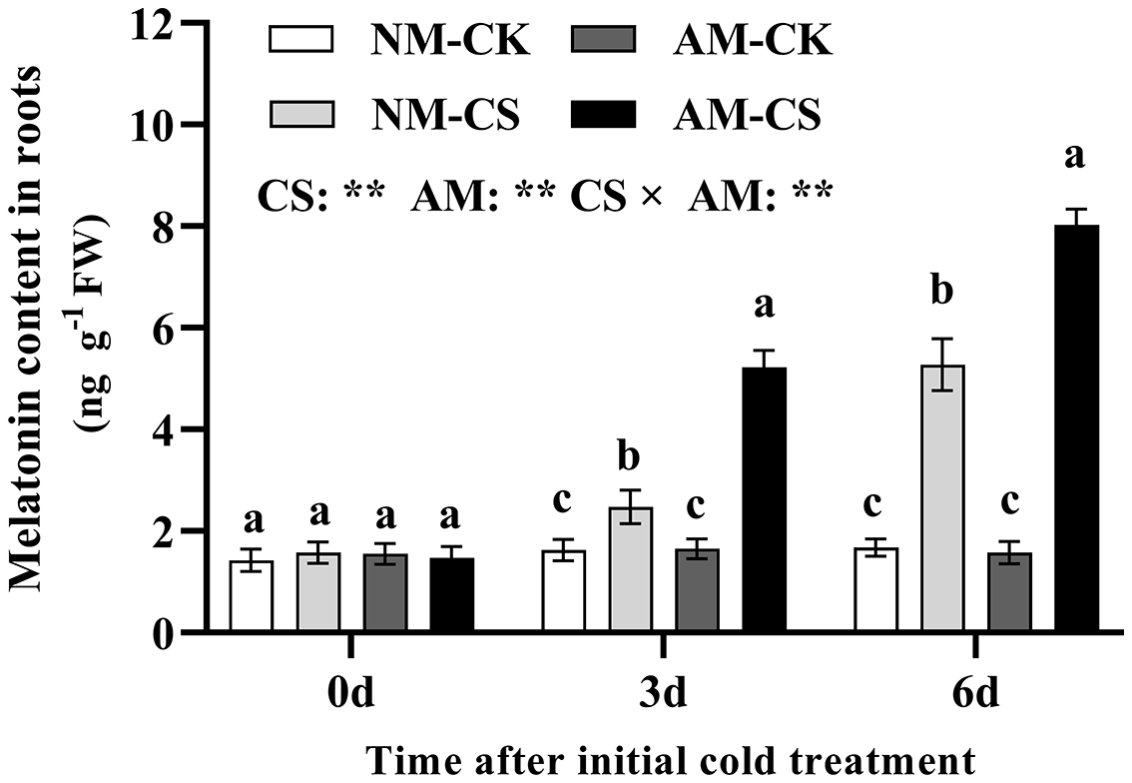
The melatonin content of mycorrhizal roots under cold stress. The data are presented as the mean ± standard deviation (*n* = 4). Different letters above the columns indicate significant differences among the means, determined by Fisher’s LSD test (*p* < 0.05). AM, AM inoculation; NM, non-AM inoculation; CK, optimal temperature condition; CS, cold stress. Significant effect of two-way ANOVA: “*,” “**,” and “***” indicate significant differences at *p* < 0.05, *p* < 0.01, and *p* < 0.001, respectively, and “ns” represents no significant difference.

Arbuscular mycorrhizal (AM) inoculation did not affect the melatonin levels in the roots of the plants in optimal temperature conditions. After 6 days of low-temperature exposure, the levels of melatonin in the roots of AM plants were 3.15 times and 4.94 times higher than those in NM plants. Cold stress significantly increased (*p* < 0.05) root melatonin levels in both NM and AM plants compared to those under optimal temperature conditions. Additionally, in perennial ryegrass maintained under cold stress, the melatonin levels were significantly higher after AM inoculation than that in the NM plants (*p* < 0.05; Figure 1). Cold stress and AM inoculation significantly affected (*p* < 0.01) the melatonin levels in the roots.

### 3.2. Expression of melatonin synthesis genes

**Figure 2:**
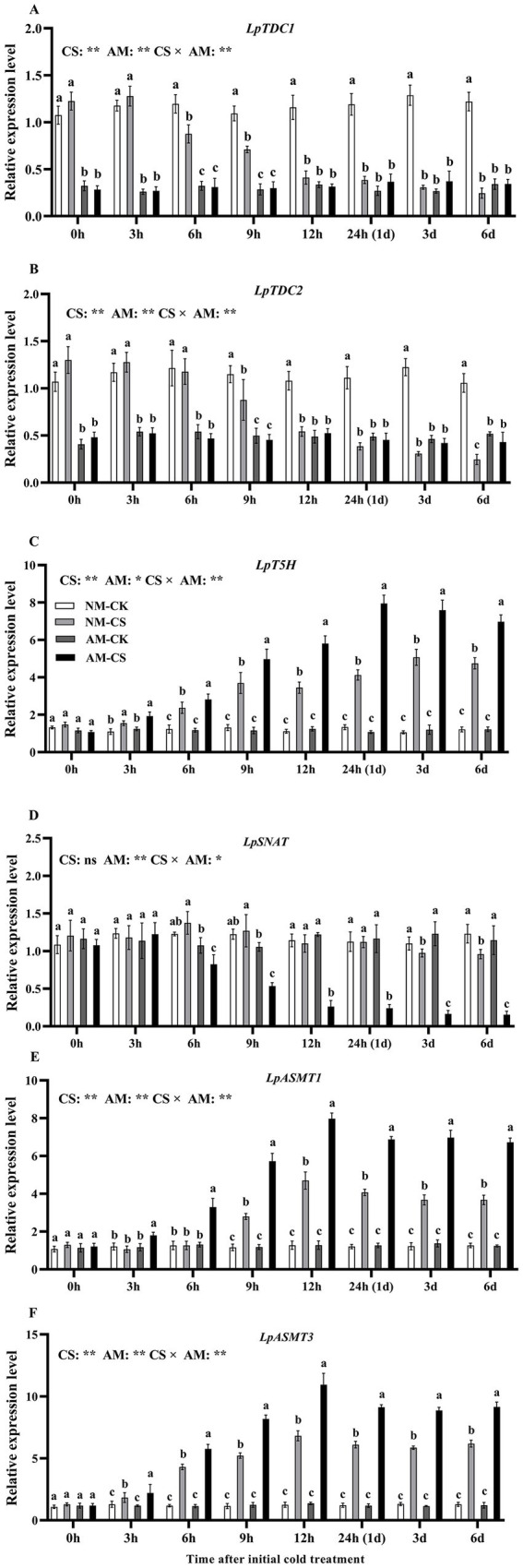
The relative expression of *LpTDC1* **(A)**, *LpTDC2* **(B)**, *LpT5H* **(C)***, LpSNAT* **(D)***, LpASMT1* **(E)**, and *LpASMT3* **(F)** in AM roots under cold stress. AM, AM inoculation; NM, non-AM inoculation; CK, optimal temperature condition; CS, cold stress. The data are presented as the mean ± standard deviation (*n* = 4). Different letters above the columns indicate significant differences among the means, determined by Fisher’s LSD test (*p* < 0.05); “*” and “**” indicate significant differences at *p* < 0.05 and *p* < 0.01, respectively.

Arbuscular mycorrhizal (AM) inoculation led to a significant decrease (*p* < 0.05) in *LpTDC1* and *LpTDC2* levels in plants grown under optimal temperatures at all time points, as demonstrated by Figures 2A,B. For NM plants, cold stress significantly downregulated (*p* < 0.05) *LpTDC1* and *LpTDC2* levels from 6 h after exposure to the low-temperature condition and significantly reduced (*p* < 0.05) *LpSNAT* levels after 3 and 6 days of exposure to the low-temperature condition. In contrast, cold stress significantly upregulated (*p* < 0.05) *LpASMT1* and *LpASMT3* levels from 9 h after exposure to the low-temperature condition.

Cold stress significantly increased (*p* < 0.05) the *LpT5H* gene level in AM roots from 3 h after exposure to low-temperature conditions, peaking at 24 h (Figure 2C). Under optimal temperature conditions, AM inoculation did not affect the relative expression of *LpSNAT*, *LpASMT1,* and *LpASMT3* at any time point (*p* > 0.05; Figures 2D–F). AM inoculation significantly increased (*p* < 0.05) the relative levels of *LpASMT1* and *LpASMT3* under optimal temperature conditions from 3 h after exposure to the low-temperature condition, which peaked at 12 h. Cold stress exposure resulted in a 6.17-fold and 8.04-fold increase in gene expression levels of *LpASMT1* and *LpASMT3*, respectively, compared to the non-stress condition. On the other hand, the expression of *LpSNAT* significantly decreased (*p* < 0.05) in the AM roots from 6 h after cold stress induction (Figure 2C). The levels of *LpASMT1* and *LpASMT3* at 3 and 6 days of cold stress had a positive association (*LpASMT1*: r = 0.879, *p* < 0.001; *LpASMT3*: r = 0.978, *p* < 0.001) with the root melatonin levels (Supplementary Figures S1, S2).

### 3.3. Arbuscular mycorrhizal colonization

**Figure 3:**
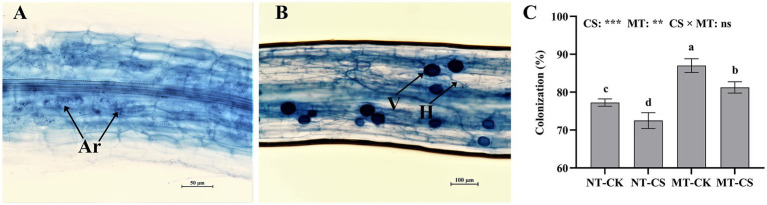
Photomicrographs of structural colonization of *Rhizophagus irregularis* in roots **(A,B)** and colonization **(C)** in mycorrhizal perennial ryegrass under cold stress. Ar, Arbuscule; V, Vesicles; H, Hypha; CK, optimal temperature condition; CS, cold stress; MT, melatonin application; NT, non-melatonin application. Different letters above the columns indicate significant differences among the means, determined by Fisher’s LSD test (*p* < 0.05). “**” and “***” indicate significant differences at *p* < 0.01 and *p* < 0.001, respectively, and “ns” represents no significant difference.

The morphological characteristics of AMF, including arbuscules, hypha, and vesicles (Figures 3A,B), were analyzed in the fine roots of the mycorrhizal perennial ryegrass. The application of melatonin significantly increased (*p* < 0.05) the arbuscular mycorrhizal (AM) colonization in perennial ryegrass roots by 16.35 and 21.04% with and without cold stress, respectively. In contrast, cold stress significantly decreased (*p* < 0.05) *Rhizophagus irregularis* colonization in roots by 11.23 and 12.31%, with and without melatonin application, respectively (Figure 3C). The AM plants to which melatonin was applied had the highest colonization of up to 89.65%.

### 3.4. Plant growth parameters

**Figure 4:**
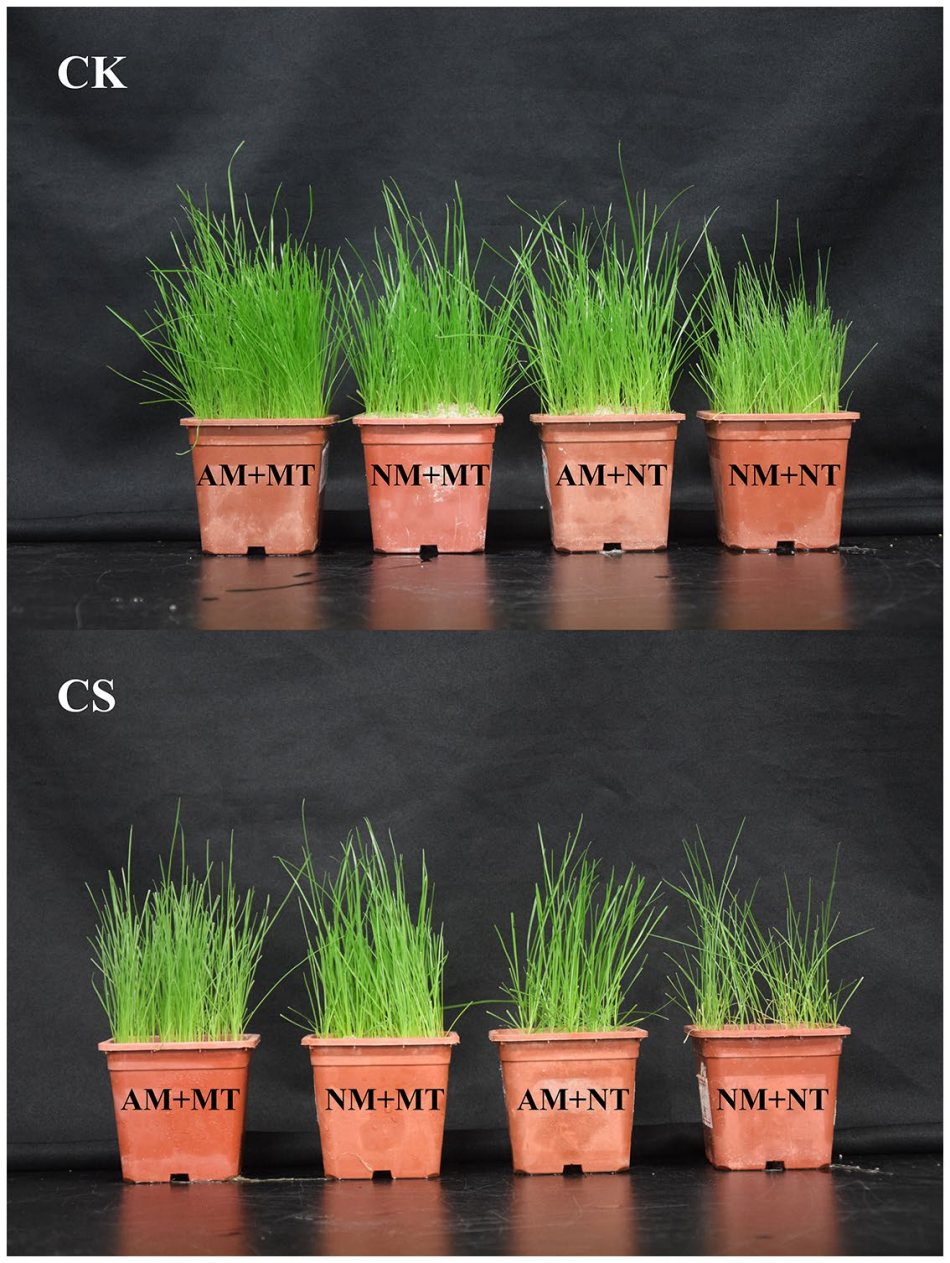
Morphological differences in different treatments. CK, optimal temperature condition; CS, cold stress; AM, AM inoculation; NM, non-AM inoculation; MT, melatonin application; NT, non-melatonin application.

Cold stress inhibited growth in perennial ryegrass, whereas single AM inoculation or exogenous melatonin application and their combined use alleviated cold stress-induced growth inhibition (Figure 4). Arbuscular mycorrhizal (AM) inoculation significantly increased (*p* < 0.05) the shoot height and the shoot and root biomass in perennial ryegrass (Table 1) relative to those of the NM plants, irrespective of the temperature conditions. Under cold stress, both NM and AM perennial ryegrass exhibited significant increases (*p* < 0.05) in shoot height and shoot and root biomass upon melatonin treatment. Specifically, NM perennial ryegrass showed a 12.63% increase in shoot height and a 39.72% and 28.57% increase in shoot and root biomass, respectively. On the other hand, AM perennial ryegrass showed an 11.88% increase in shoot height and a 22.32% and 21.73% increase in shoot and root biomass, respectively. The shoot height and the shoot and root biomass of cold-stressed plants were significantly lower than those not exposed to stress (*p* < 0.05).

**Table 1:** Growth parameters, including shoot and root dry weight (DW), shoot height, net photosynthetic rate (Pn), and photochemical efficiency (*Fv/Fm*) of perennial ryegrass inoculated with/without the AM fungus *Rhizophagus irregularis* under cold stress and after melatonin application.

Treatments		Shoot DW (g plant^−1^)	Root DW (g plant^−1^)	Shoot height (cm)	Pn	Fv/Fm
Melatonin	NM-CK	1.81 ± 0.14bc	0.41 ± 0.04bc	15.52 ± 0.04b	11.33 ± 1.04a	0.85 ± 0.04a
	NM-CS	1.02 ± 0.12e	0.21 ± 0.03e	14.16 ± 0.04e	7.26 ± 0.74c	0.53 ± 0.02c
	AM-CK	2.19 ± 0.21a	0.59 ± 0.04a	17.12 ± 0.04a	11.50 ± 1.12a	0.83 ± 0.04a
	AM-CS	1.17 ± 0.17d	0.28 ± 0.03d	14.82 ± 0.04d	8.57 ± 0.71b	0.66 ± 0.02b
Non-melatonin	NM-CK	1.71 ± 0.14c	0.34 ± 0.04c	15.13 ± 0.04c	10.56 ± 0.81a	0.78 ± 0.04a
	NM-CS	0.73 ± 0.12f	0.16 ± 0.02f	13.17 ± 0.04f	6.06 ± 0.54d	0.44 ± 0.03d
	AM-CK	1.91 ± 0.18b	0.51 ± 0.03b	15.97 ± 0.04b	11.02 ± 0.72a	0.83 ± 0.06a
	AM-CS	0.91 ± 0.16e	0.23 ± 0.03de	14.07 ± 0.04e	7.51 ± 0.61c	0.56 ± 0.04c
ANOVA						
CS		**	**	**	**	**
AM		**	**	*	*	*
MT		***	**	*	*	*
CS × AM × MT		*	*	ns	ns	ns

Difference in various growth parameters and comparative analysis. The data are the means ± standard deviation (*n* = 4). AM, AM inoculation; NM, non-AM inoculation; CK, optimal temperature condition; CS, cold stress. Different letters means significantly different based on *p* < 0.05. “*,” “**,” “***” indicate significant differences at *p* < 0.05, *p* < 0.01, and *p* < 0.001, respectively, and “ns” represents no significant difference. MT, melatonin application.

The net photosynthetic rate (Pn) and photochemical efficiency (*Fv/Fm*) of perennial ryegrass were significantly lower in cold-stressed plants than that in non-stressed plants (*p* < 0.05). Under cold stress conditions, the application of melatonin led to a significant increase (*p* < 0.05) in both net photosynthetic rate (Pn) and photochemical efficiency (*Fv/Fm*) in non-mycorrhizal (NM) perennial ryegrass, with an increase of 20.01% and 18.18%, respectively. Similar results were observed in NM perennial ryegrass, with an increase of 28.58% and 17.85%, respectively. AM-inoculated plants with exogenously applied melatonin showed significantly higher Pn and *Fv/Fm* compared to the plants without melatonin application under cold stress (*p* < 0.05).

### 3.5. Antioxidant activity

**Figure 5:**
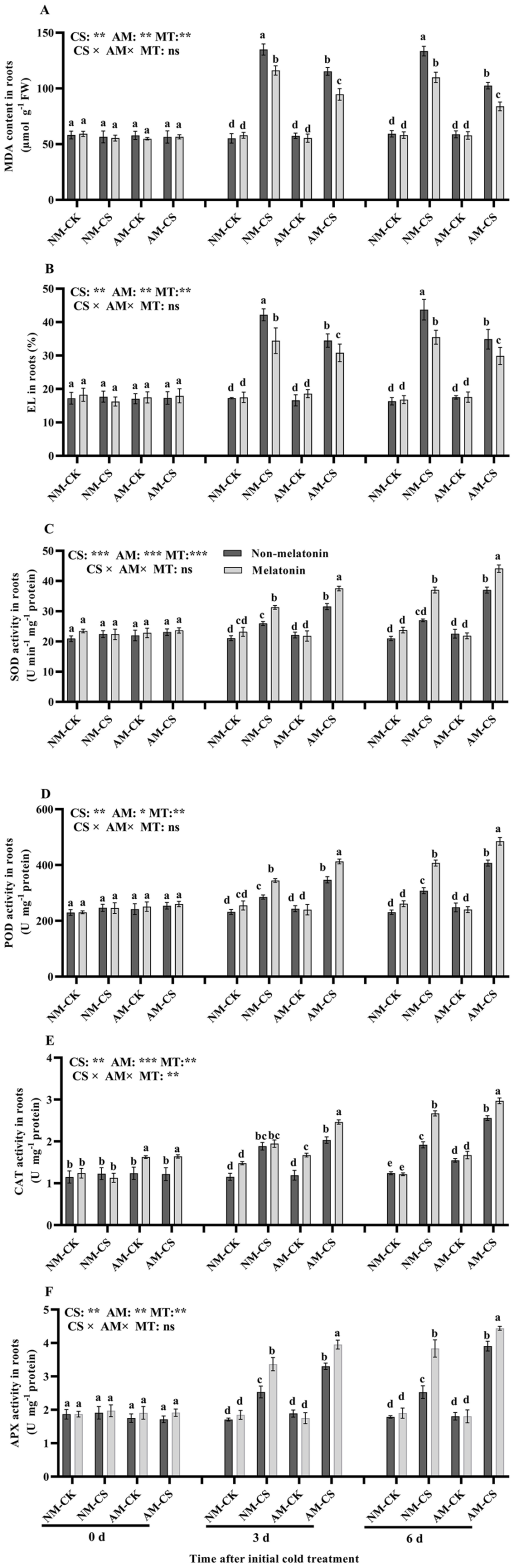
The malonaldehyde (MDA) contents **(A)**, electrolyte leakage (EL) **(B)**, superoxide dismutase (SOD) activity **(C)**, peroxidase (POD) activity **(D)**, catalase (CAT) activity **(E)**, and ascorbate peroxidase (APX) activity **(F)** in AM roots under cold stress and after melatonin application. AM, AM inoculation; NM, non-AM inoculation; CK, optimal temperature condition; CS, cold stress. Different letters above the columns indicate significant differences among means, determined by Fisher’s LSD test (*p* < 0.05); “*,” “**,” and “***” indicate significant differences at *p* < 0.05, *p* < 0.01, and *p* < 0.001, respectively, and “ns” represents no significant difference. MT, melatonin application.

The MDA levels and EL were significantly higher (*p* < 0.05) in the roots of cold-stressed plants compared to that in the roots of non-stressed plants (Figures 5A,B). Melatonin application remarkably decreased the MDA levels and EL in the cold-stressed AM and NM plants (*p* < 0.05). However, it did not influence the MDA levels and EL in the non-stressed plants (*p* > 0.05). Neither AM inoculation nor melatonin application affected the root SOD, POD, and APX activities based on optimal temperature situations (*p* > 0.05; Figures 5C,D,F). However, AM inoculation combined with melatonin application enhanced the root SOD, POD, CAT, and APX activities in cold-stressed plants. On day 6, in optimal temperature situations, AM inoculation increased the root CAT activity by 24.19% and 31.13% in the presence and absence of melatonin application, respectively (Figure 5E, *p* < 0.05). Cold-stressed AM-inoculated roots with exogenously applied melatonin recorded the highest SOD, POD, APX, and CAT activities on days 3 and 6, among all treatments.

### 3.6. Proline level and production

**Figure 6:**
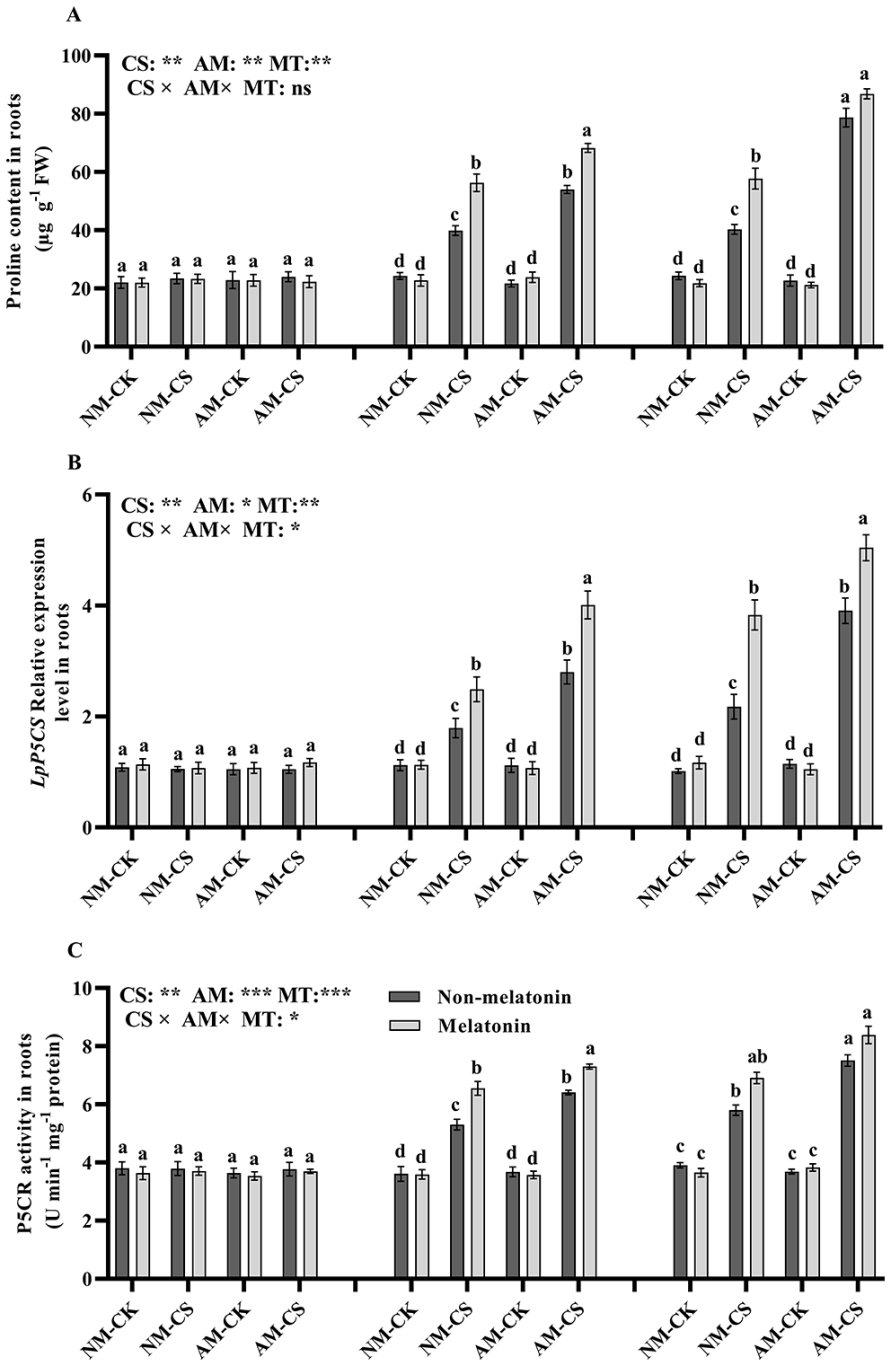
The proline content **(A)**, *LpP5CS* expression **(B)**, and P5CR activity **(C)** in AM roots under cold stress and melatonin application. AM, AM inoculation; NM, non-AM inoculation; CK, optimal temperature condition; CS, cold stress. Different letters above the columns indicate significant differences among the means, determined by Fisher’s LSD test (*p* < 0.05); “*,” “**,” and “***” indicate significant differences at *p* < 0.05, *p* < 0.01, and *p* < 0.001, respectively, and “ns” represents no significant difference. MT, melatonin application.

Melatonin application did not have a significant impact (*p* > 0.05) on proline levels (Figure 6A), P5CR activity (Figure 6B), or *LpP5CS* transcription (Figure 6C) in AM-inoculated roots when compared to no melatonin application on days 0, 3, and 6 under optimal temperature conditions. However, Cold stress increased the proline level, P5CR activity, and the transcription of *LpP5CS* in the roots. On day 3 after low-temperature treatment, melatonin application significantly increased (*p* < 0.05) proline level, P5CR activity, and *LpP5CS* level (*p* < 0.05) by 40.13%, 22.64%, and 39.10%, respectively, in NM plants and by 25.92%, 16.39%, and 42.85%, respectively. However, on day 6, melatonin did not significantly affect the proline level and P5CR activity in AM plants.

### 3.7. PAL and PPO activities

**Figure 7:**
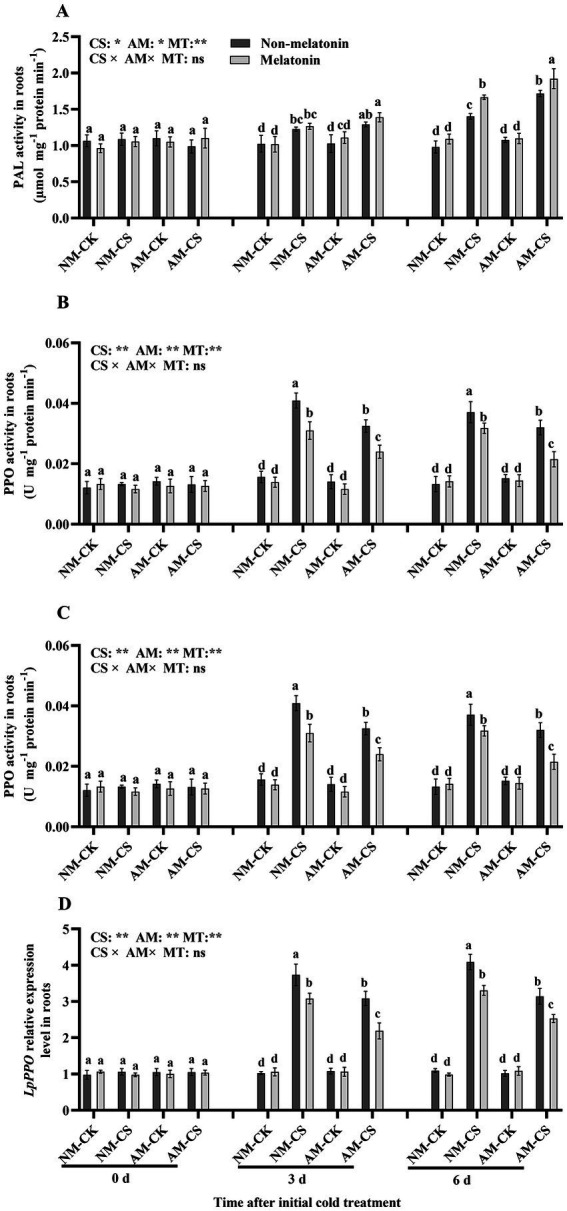
The phenylalanine ammonia-lyase (PAL) activity **(A)**, *LpPAL* expression **(B)**, polyphenol oxidase (PPO) activity **(C)**, and *LpPPO* expression **(D)** in AM roots under cold stress and melatonin application. AM, AM inoculation; NM, non-AM inoculation; CK, optimal temperature condition; CS, cold stress. Different letters above the columns indicate significant differences among means, determined by Fisher’s LSD test (*p* < 0.05); “*,” “**,” and “***” indicate significant differences at *p* < 0.05, *p* < 0.01, and *p* < 0.001, respectively, and “ns” represents no significant difference. MT = melatonin application.

Under optimal temperature conditions, AM inoculation and melatonin application did not significantly influence (*p* > 0.05) PAL and PPO activities (Figures 7A,C). They also did not influence the transcription of *LpPAL* and *LpPPO* (Figures 7B,D). However, the activity of PAL and PPO was significantly elevated (*p* < 0.05) on days 3 and 6, after low-temperature treatment. The plants inoculated with AM fungi and treated with melatonin had the highest PAL activity and *LpPAL* expression among all treatments on days 3 and 6 after being exposed to cold stress, with values of 1.92 μmol mg^−1^ protein min^−1^ and 2.81, respectively.

### 3.8. Free phenolics, total flavone, starch, and soluble sugar contents

**Figure 8:**
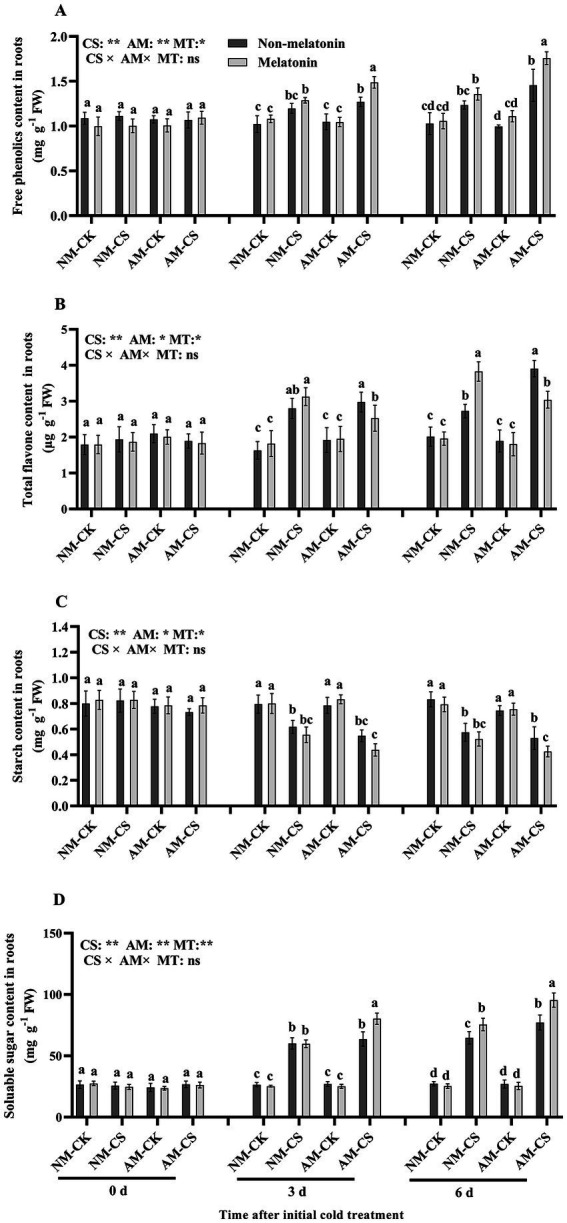
The free phenolic content **(A)**, total flavone content **(B)**, starch content **(C)**, and soluble sugar content **(D)** in AM roots under cold stress and after melatonin application. AM, AM inoculation; NM, non-AM inoculation; CK, optimal temperature condition; CS, cold stress. Different letters above the columns indicate significant differences among the means, determined by Fisher’s LSD test (*p* < 0.05); “*,” “**,” and “***” indicate significant differences at *p* < 0.05, *p* < 0.01, and *p* < 0.001, respectively, and “ns” represents no significant difference. MT = melatonin application.

Cold stress increased (*p* < 0.05) the free phenolic content in the roots of perennial ryegrass. Melatonin application did not significantly affect (*p* > 0.05) its content in the NM plants but significantly increased the free phenolic content in the AM plant roots 3 and 6 days after exposure to cold stress (*p* < 0.05; Figure 8A). Cold stress significantly increased (*p* < 0.05) total flavone content on days 3 and 6 in all treatment. The AM plants treated with melatonin exhibited a significant decrease (*p* < 0.05) in total flavone content by 13.79% and 23.07% on days 3 and 6 after low-temperature treatment, respectively, compared to the non-melatonin-treated plants (Figure 8B). Furthermore, melatonin application significantly reduced (*p* < 0.05) the starch content in the roots of AM plants by 26.19% compared to those without melatonin treatment on day 6. However, there was no significant effect on the starch content in NM plants at any time point after low-temperature treatment. Cold stress significantly decreased (*p* < 0.05) starch content in AM and NM plants (Figure 8C). In roots, melatonin application significantly increased (*p* < 0.05) the soluble sugar content by 26.62% in AM-inoculated plants compared to NM plants on day 3 under low-temperature conditions and in AM and NM plants on day 6 after exposure to low temperatures, compared to that in plants non-treated with melatonin. However, melatonin treatment did not affect the soluble sugar content in the NM roots 3 days after exposure to cold stress (Figure 8D). The roots of AM-inoculated and melatonin-treated plants 3 and 6 days after exposure to cold stress exhibited the highest free phenolic, total flavone, and soluble sugar content among all treatments.

### 3.9. Principal component analysis

**Figure 9:**
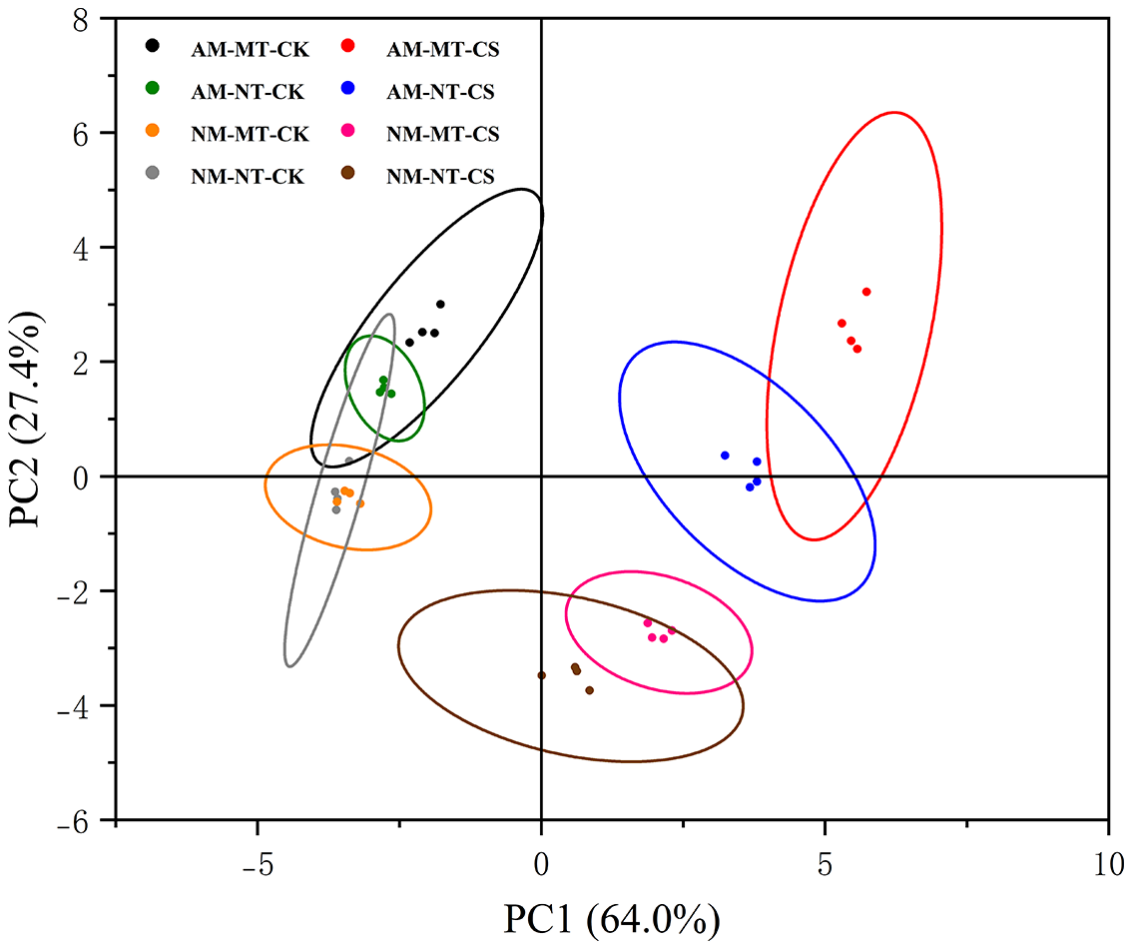
The principal component analysis of antioxidant-related and protecting molecules-related parameters in the roots of perennial ryegrass inoculated with/without the AM fungus *Rhizophagus irregularis* under cold stress. The analysis was conducted using data for all antioxidant-related parameters (MDA, EL, SOD, POD, CAT, and APX) and protective molecule-related parameters (proline, free phenolic, total flavone, starch, and soluble sugar content; P5CR, PAL, and PPO activity; *LpP5CS, LpPAL,* and *LpPPO* expression) in roots. Each point represents a type of treatment in this experiment. AM-MT-CK, Mycorrhizal plants administered melatonin under optimal temperature conditions; AM-NT-CK, Mycorrhizal plants not administered melatonin under optimal temperature conditions; NM-MT-CK, Non-mycorrhizal plants administered melatonin under optimal temperature conditions; NM-NT-CK, Non-mycorrhizal plants not administered melatonin under optimal temperature conditions; AM-MT-CS, Mycorrhizal plants administered melatonin under cold stress; AM-NT-CS, Mycorrhizal plants not administered melatonin under cold stress; NM-MT-CS, Non-mycorrhizal plants administered melatonin under cold stress; NM-NT-CS, Non-mycorrhizal plants not administered melatonin under cold stress.

The principal component analysis (PCA) of antioxidant-related and protective molecule-related parameters in AM/NM roots showed the effects of cold stress and melatonin application (Figure 9). In roots, PC1 covered 64.0% of the variance and PC2 covered 27.4% of the variance. Treatment with AM-MT-CS was the most different from other treatments. Moreover, the samples treated with AM-MT-CS are located in the upper right corner of the figure, indicating that under cold stress, the combined application of *R. irregularis* and melatonin is more effective in enhancing antioxidant activity and osmotic regulation capacity compared to other treatments. Melatonin application and non-melatonin application treatment as well as optimal temperature and low temperature conditions did not cluster in the same category.

## 4. Discussion

Melatonin acts as an antioxidant and a signaling agent in turfgrass plants (Alam et al., 2018). It has been reported that melatonin helps to protect organelles from oxidative stress caused by abiotic stress, such as drought or heat stress (Arnao and Hernandez-Ruiz, 2019; Sun et al., 2021). Our findings suggest that cold stress can enhance melatonin synthesis in plant roots, which may promote antioxidant activity and provide protection against low-temperature stress. AM inoculation promoted melatonin synthesis in the roots of cold-stressed perennial ryegrass. These results suggested that cold-stressed AM-inoculated plants accumulate abundant levels of melatonin when exposed to cold-induced oxidative stress. The root-derived endophytic bacteria from the grapevine (*Vitis quinquangularis*) can generate melatonin under abiotic stress (Jiao et al., 2016). According to Liu et al. (2016), *Trichoderma asperellum* fungi might accumulate melatonin in response to abiotic stress. Also, in this study, due to cold stress, the melatonin generated by the AMF *R. irregularis* might have facilitated melatonin accumulation within perennial ryegrass roots. Melatonin production efficiency depends on serotonin levels, which helps in determining the amount of melatonin accumulated in plants (Kang et al., 2013; Byeon et al., 2014). In salt-stressed sunflower (*Helianthus annuus* L) plants, root melatonin concentration was found to increase due to the increase in ASMT activity, which indicated that ASMT promoted melatonin production (Mukherjee et al., 2014). *LpASMT1* and *LpASMT3* levels showed a significantly positive correlation with melatonin levels. According to Kang et al. (2013), ASMT, but not SNAT, might limit the rate of melatonin generation in plants. The cadmium-stressed rice (*Oryza sativa*) plants with lower expression of SNAT and higher expression of ASMT accumulated a greater amount of melatonin (Byeon et al., 2015). The stimulation of *LpASMT1* and *LpASMT3* expression through AM inoculation probably induced melatonin production in the roots. As AM-inoculated plants have greater control over melatonin, they can better withstand low-temperature stress compared to NM plants.

The growth parameters were significantly affected by exposure to low temperatures, which may be due to a reduction in Pn and *Fv/Fm*. This phenomenon can be attributed to several factors such as compromised CO_2_ fixation, disturbance in water balance, and decline in nutrient uptake and metabolic activities. Several previous studies on cold stress in plants have also reported similar observations (Waraich et al., 2012; Ihtisham et al., 2023). The reduction in leaf photochemical parameters under low-temperature conditions could be attributed to damage and loss of PSII reaction centers, and disruptions in electron transport that are necessary for reaction center excitation, as previously reported (Baker, 2008). The findings of this study showed that cold stress significantly decreased the root mycorrhizal colonization of perennial ryegrass. Such reduced AMF colonization was probably associated with a decrease in the photosynthesis of plants under cold stress; thus, decreasing fungal carbon allocation, as indicated in the study of Smith and Read (2008). Similar results were found in a study on cold-stressed plants of cucumber (*Cucumis sativus*; Ma et al., 2015) and maize (Li et al., 2020). In this study, melatonin treatment and AM inoculation enhanced plant development in the cold-stressed perennial ryegrass, as determined by an increase in the shoot height and the shoot and root biomass. Both AM symbiosis and melatonin protected the photosynthetic system of the host plant and increased its photosynthetic rate under cold stress, which increased the biomass, improved carbon allocation to AMF, and enhanced the colonization rate.

Generally, ROS levels in plant cells under suitable growth conditions are in a dynamic state of balance (Baxter et al., 2013). Cold stress can induce excessive ROS generation and peroxidation of membrane lipids, resulting in the dysfunction of plant metabolism (Sies and Jones, 2020). EL and MDA levels were determined by evaluating the oxidative stress levels of plants (Chang et al., 2018). AM inoculation and melatonin treatment decreased the EL and MDA levels to protect perennial ryegrass against oxidative stress. The O_2_^•−^/H_2_O_2_ system enzymatically converts ROS into harmless products such as H_2_O, making it crucial for managing oxidative stress (Zhang et al., 2016). In plants, the active oxygen scavenging system mostly contains antioxidant molecules along with antioxidant enzymes, including SOD, APX, and POD (Chen et al., 2020). SOD catalyzes the O_2_^•−^ disproportion to H_2_O_2_, whereas CAT, APX, and POD help in eliminating H_2_O_2_ (Zhang et al., 2019). Thus, AM inoculation combined with melatonin treatment facilitated cold resistance, which was probably correlated with increased antioxidant enzyme activity and decreased oxidative stress.

Proline strongly regulates redox potentials, protecting macromolecules from denaturation and scavenging by hydroxyl radicals (Latef et al., 2016). Additionally, proline maintains water homeostasis during cold stress (Hajiboland et al., 2019). It has been found that proline accumulation in plants can enhance their freezing and frost resistance and their winter survival, as shown by studies on proline-accumulating barley (*Hordeum vulgare* L.) lines (Tantau et al., 2004). The enzymes P5CS and P5CR are crucial in the proline synthesis pathway in plants (Jiang et al., 2023). In *Medicago sativa*, the application of melatonin has been shown to increase proline accumulation and production, which helps to repair molecular damage caused by drought (Antoniou et al., 2017). Zhang et al. (2020) found that melatonin promoted proline production by upregulating *MtP5CS* and increasing P5CR activity to alleviate Pb stress in *Medicago truncatula*. Melatonin treatment increased the proline level under cold stress conditions, which indicated that melatonin mitigated cold stress in the roots of AM and NM perennial ryegrass.

PAL is a key enzyme that participates in the phenylpropanoid metabolic pathway. The metabolites of the phenylpropanoid pathway include polyphenols, flavonoids, and anthocyanidins (Zhang and Liu, 2015). Low temperatures trigger the synthesis and buildup of protective molecules to mitigate the effects of cold stress. Phenol oxidation by PPO generates highly harmful quinones under cold stress. Furthermore, PPO activity lowers antioxidant activity and membrane integrity, leading to membrane lipid peroxidation (Habibi et al., 2021). Oxidation and polymerization can adversely affect the properties of phenolic substances and hinder their ability to scavenge ROS (Rice-Evans et al., 1996). A decrease in enzymatic processes (via PPO) related to an increase in the free production of phenolics (via PAL) and accumulation under cold stress can strongly promote cold resistance in *L. perenne*. PAL can be activated at suboptimal temperatures, while oxidizing enzymes (PPO) might be suppressed, which is the acclimation mechanism for overcoming cold stress (Habibi et al., 2022). Inoculation with *R. irregularis* or application of melatonin promoted phenolic metabolism and improved the ability of perennial ryegrass to cope with the effects of cold stress. Melatonin treatment enhanced PAL activity in the kiwifruit (*Actinidia chinensis*) and improved its cold tolerance, thus, indicating that melatonin might be associated with flavonoid production (Jiao et al., 2022). An increase in PAL activity, along with the accumulation of phenolics, often occurs during plant biotic interactions (Derksen et al., 2013) involving AMF inoculation (Hajiboland et al., 2019). Additionally, the upregulated phenylpropanoid pathway might be involved in specific host–AMF signaling. Alternatively, it might also reflect the plant defense strategy (García-Garrido and Ocampo, 2002).

Starch is a crucial molecule that plays a role in regulating plant responses to abiotic stress, such as extreme temperature, high salinity stress, and water deficit (Thalmann and Santelia, 2017). The findings of this study revealed a significant reduction in starch concentration under low-temperature conditions, while the concentration of soluble sugars increased significantly. In such challenging environments, when photosynthesis might be restricted, plants break down starch to obtain energy and carbon. An increase in the soluble sugar content during cold stress may protect the cells by playing the role of osmoprotectants or compatible solutes (Shah et al., 2021). Higher soluble sugar concentrations in AM plants were accompanied by lower starch concentrations, which suggested that starch degradation in plants was stimulated by AMF inoculation under cold stress. In contrast, Pasbani et al. (2020) found that higher soluble carbohydrate concentration in mycorrhizal eggplants (*Solanum melongena* L.) was not accompanied by lower starch concentrations under low-temperature stress. The authors suggested that the decrease in starch concentration observed in this study was likely due to a reduction in plant development and a decline in sink strength caused by cold stress. The increase in soluble sugar content may be attributed to the role of sugars in maintaining membrane hydration and ROS scavenging under low-temperature stress. Liang et al. (2018) reported that melatonin treatment can enhance antioxidant capacity and flavonoid production by increasing SOD, POD, and CAT activities and alleviating leaf senescence. Exogenous melatonin can alleviate plant cold stress by accelerating starch degradation while increasing phenolics, proline, and soluble sugar contents, indicating that melatonin improves plant osmoregulation (Hu et al., 2016; Zhang et al., 2021). Thus, melatonin treatment can enhance plant growth and antioxidant ability by accumulating protective molecules and triggering antioxidant activity in perennial ryegrass under cold stress. No significant additive effect on the accumulation of protective molecules, such as proline and flavonoids, was observed under cold stress by the combination of AM inoculation and exogenous melatonin application. The AM fungus and melatonin application might enhance cold tolerance in ryegrass through different pathways. The use of melatonin can significantly influence the levels of plant hormones that are involved in coping with abiotic stress (Arnao and Hernández-Ruiz, 2017). This includes the production of flavonoids and proline, both of which are controlled by salicylic acid (Ben Abdallah et al., 2016). Additional studies are required to determine the effects of melatonin-phytohormone interactions on abiotic stress resistance in AM plants.

## 5. Conclusion

AM inoculation can trigger melatonin accumulation by upregulating the levels of *LpASMT1* and *LpASMT3* in roots. Melatonin treatment promoted AM symbiosis in the roots of perennial ryegrass under cold stress. Plant growth and stress tolerance ability were improved by AM inoculation and melatonin application. The positive effect of the AM symbiosis, reduction in oxidative damage, increase in antioxidant enzyme activity, and accumulation of protective molecules may collectively contribute to this synergy. Overall, the findings of this study indicated that melatonin treatment could improve the antioxidant activity, AM symbiosis, and accumulation of protective molecules; thus, promoting mycorrhizal plant development and cold tolerance. Therefore, the combination of melatonin treatment and AM inoculation can be a potential strategy to cultivate host plants in regions with severe winter temperatures.

## Data availability statement

The original contributions presented in the study are included in the article/Supplementary material, further inquiries can be directed to the corresponding authors.

## Author contributions

HW, JW, QW, WHe, SL, JH, WHu, MT, and HC made contributions to the study conception and design and commented on former versions of the paper. HC and MT conceived and initiated the project. All the experiments were performed by HW and JW. QW, JH, and HW were responsible for conducting the material preparation and data collection. The data analysis was carried out by WHe and SL. HW was responsible for writing the first draft of the manuscript. WHu reviewed and edited the manuscript. All authors contributed to the article and approved the submitted version.

## Funding

This research was funded by the Laboratory for Lingnan Modern Agriculture Project (project number: NZ2021025) and the National Natural Science Foundation of China (project number: 32071639).

## Conflict of interest

The authors declare that the research was conducted in the absence of any commercial or financial relationships that could be construed as a potential conflict of interest.

## Publisher’s note

All claims expressed in this article are solely those of the authors and do not necessarily represent those of their affiliated organizations, or those of the publisher, the editors and the reviewers. Any product that may be evaluated in this article, or claim that may be made by its manufacturer, is not guaranteed or endorsed by the publisher.
