# Supplementary material for: Role of melatonin in enhancing arbuscular mycorrhizal symbiosis and mitigating cold stress in perennial ryegrass (Lolium perenne L.)
Source: Front Microbiol. 2023 May 22;14:1123632. doi: 10.3389/fmicb.2023.1123632 (PMC10239815; doi:10.3389/fmicb.2023.1123632)
Supplement: Supplementary file 1 [file Table_1.DOCX]

| **Gene Name** | **Forward primer sequence (5' - 3')** | **Reverse primer sequence(5' - 3')** |
| --- | --- | --- |
| *LpelF4A (Reference gene)* | AACTCAACTTGAAGTGTTGGAGTG | AGATCTGGTCCTGGAAAGAATATG |
| *LpASMT1* | CAGCAGCATACTACACTCATACA | TCCAGCTGACCTTGAAGTAAC |
| *LpASMT3* | CTCAAGTGGGTTATGTGTCTGT | CTACCACGGCATCGAAGATTAT |
| *LpT5H* | ATCAACACATTCGCCATGGG | AGCTTGTAGTCCGGATCCTTG |
| *LpTDC1* | TGGCCAAGATGTTCGAAGAC | TGAAGCACACGAGAGCAAAG |
| *LpTDC2* | TGCCAGTGAAGCAGTTCTTG | ATTCCTGCAATCTGGCATGC |
| *LpSNAT* | TCTTCAATATGCGGCGTCTG | ACCGTCTTTTCGCTTGCTTC |
| *LpP5CS* | TGCAAAAGCCGCAGAATGAG | ACTGTCTGTCACGAGAAGTTGG |
| *LpPAL* | GATGCTCGCAAAGAAGCTCG | TGGAAGAGATGAGGCCGAGA |
| *LpPPO* | TTCTCCTGCACTACCGCAAG | GGATCCGCTCGTGGAAGTAG |

Table S1. RT-qPCR primers used in this study.
