# Supplementary figures and images for: Role of melatonin in enhancing arbuscular mycorrhizal symbiosis and mitigating cold stress in perennial ryegrass (Lolium perenne L.)
Source: Front Microbiol. 2023 May 22;14:1123632. doi: 10.3389/fmicb.2023.1123632 (PMC10239815; doi:10.3389/fmicb.2023.1123632)

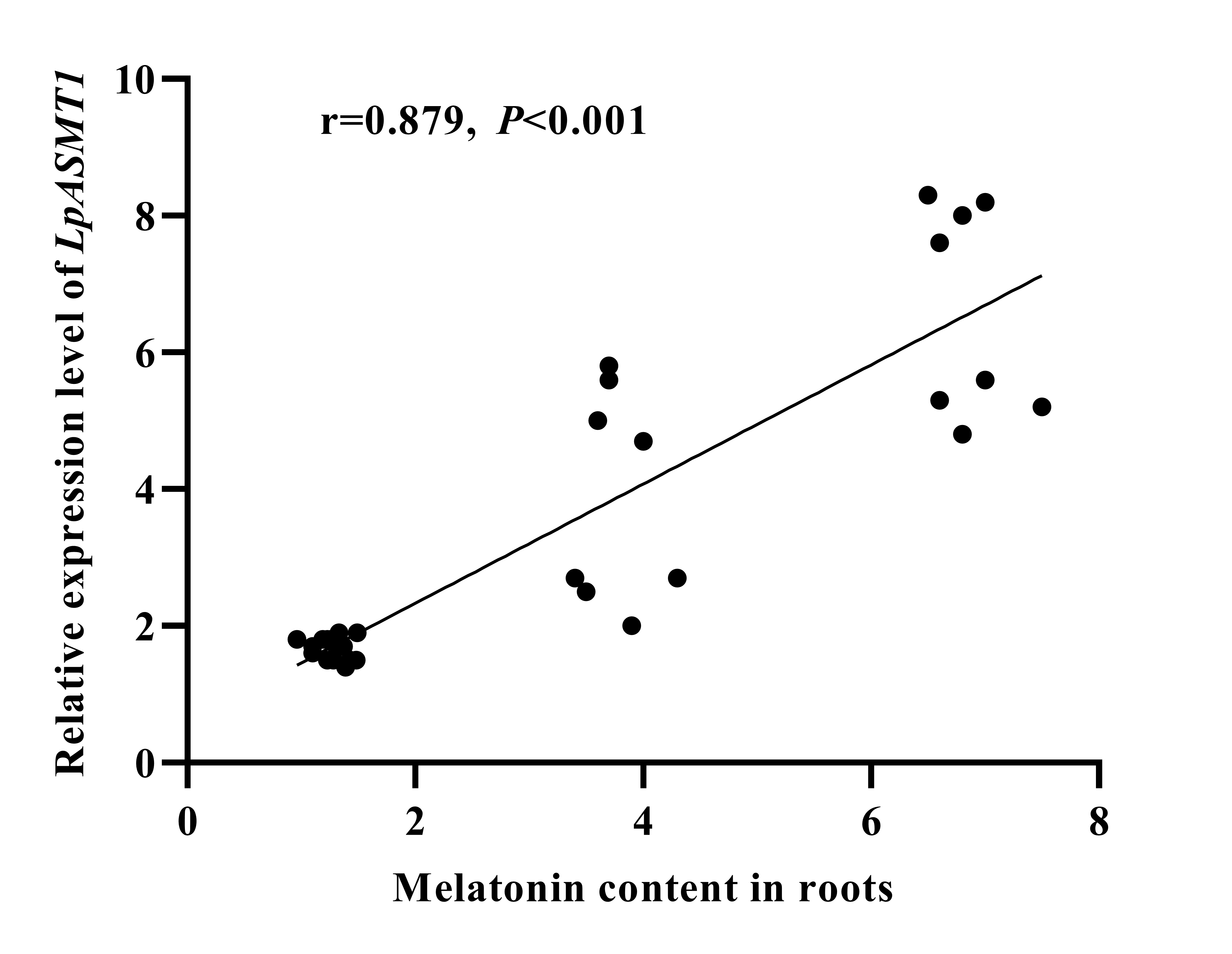

Supplement: Supplementary file 2 [file Image_1.TIF]

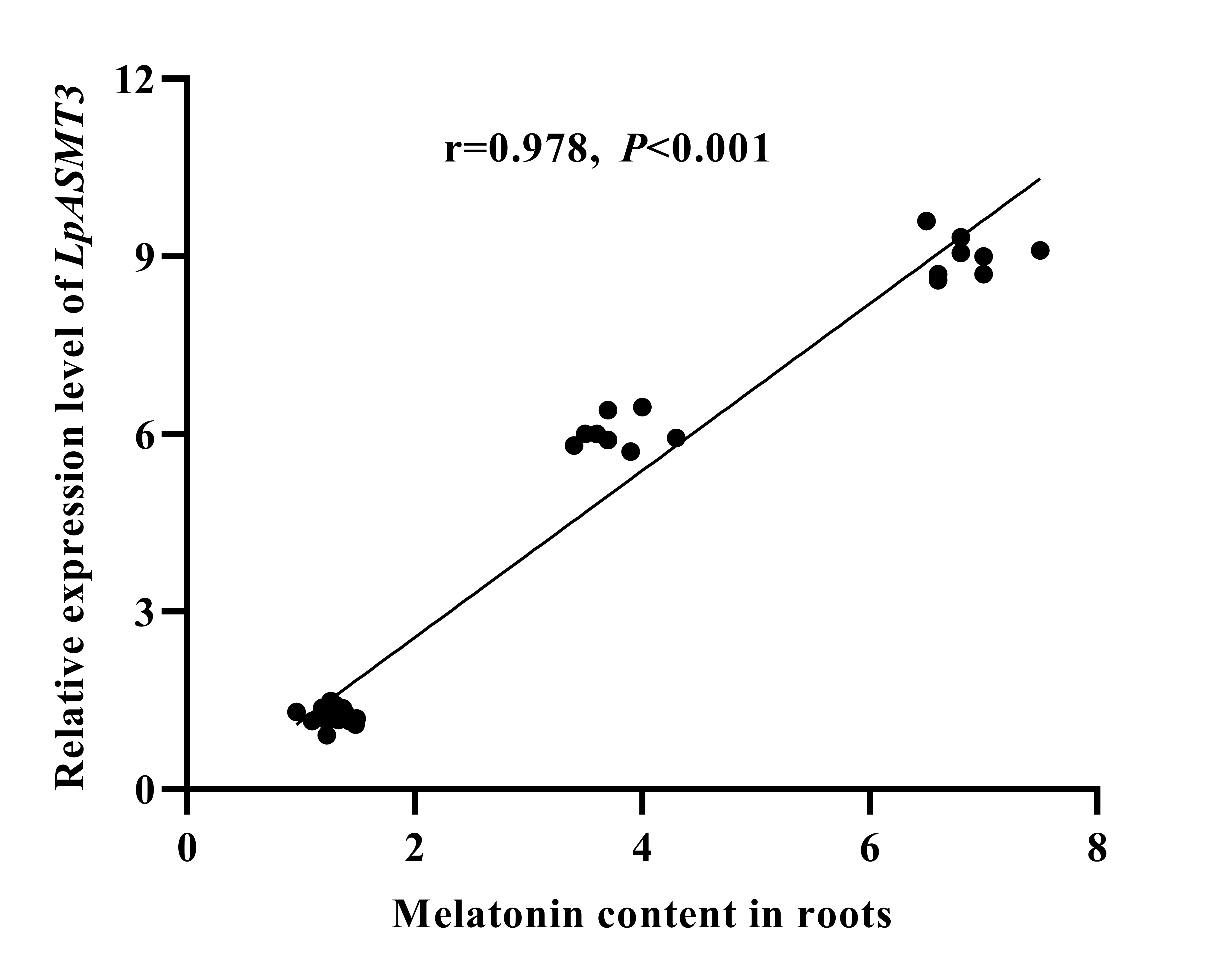

Supplement: Supplementary file 3 [file Image_2.TIF]
